# Experience as knowledge: Disability, distillation and (reprogenetic) decision-making

**DOI:** 10.1016/j.socscimed.2017.09.013

**Published:** 2017-09-08

**Authors:** Felicity K. Boardman

**Affiliations:** Division of Health Sciences, Warwick Medical School, University of Warwick, Coventry, CV4 7AL, UK

**Keywords:** United Kingdom, Experiential knowledge, Spinal Muscular Atrophy, Disability, Reproduction, Genetics, Decision-making

## Abstract

‘Experiential knowledge’ is increasingly recognised as an important influence on reproductive decision-making. ‘Experiential knowledge of disability’ in particular is a significant resource within prenatal testing/screening contexts, enabling prospective parents to imagine and appraise future lives affected by disability. However, the concept of ‘experiential knowledge’ has been widely critiqued for its idiosyncrasy, its impermanence and consequently its perceived inferiority to (medical) knowledge. This paper explores some of these key critiques of experiential knowledge through an analysis of its constitution and uses in the context of reproductive decision-making. Seventeen UK-resident women with Spinal Muscular Atrophy (SMA), or with SMA in their family, took part in two in-depth interviews: one in 2007–9 and the other in 2013–4. By comparing and contrasting these women’s accounts at two time points, this paper demonstrates the stark contrast between ‘lived experience’ of SMA (the visceral everyday realities of life with the condition) and the various way(s) this experience was transformed into, and presented as, ‘knowledge’ through the processes of making, and accounting, for reproductive decisions. The analysis highlights that multiple, distinct and sometimes competing experiential frameworks are used to conceptualise SMA across time and context. However, rather than evidence of its fallibility, this finding highlights that ‘knowledge’ is an inappropriate vessel with which to capture and transfer ‘experiential knowledge’. Rather, we need to consider how to value such insight in ways that harnesses its inherent strength without leaving it vulnerable to the epistemological critiques attracted by labelling it ‘knowledge’.

## 1 Introduction

Experiential knowledge, that is, knowledge gained through either ‘embodied’ (direct bodily experience) or ‘empathetic’ (knowledge gained through close emotional ties with others) experience of a phenomenon (Abel and Browner, 1998), has been increasingly acknowledged in the health and social science literature as a significant body of knowledge (Prior, 2003; Baillergeau and Duyvendak, 2016; Caron-Finterman et al., 2005) and one of substantial influence in the context of health care decision-making (Bulme, 2016; Lippman, 1999; Markens et al., 2010; France et al., 2011). The decline of paternalistic models of medicine, the paralleled increase in, and emphasis on, personalised medicine and patient organisations together with the gains made by both the feminist and disability rights movement since the 1970s have all contributed to the expanding value placed on the realm of the experiential as a resource with which to supplement, supplant or challenge medical knowledge (Abel and Browner, 1998; Frank, 1995; D’Agincourt-Canning, 2005; Williams and Popay, 1994; Bulme, 2016; Baillergeau and Duyvendak, 2016; Rabeharisoa et al., 2014; Britten and Maguire, 2016; Boardman, 2014).

Within the domain of reproduction, the development of increasingly sophisticated reproductive technologies (such as NIPT), and the acceleration of advances in genomic medicine have created a context in which the role and value assigned to experiential knowledge is gaining significance. Would-be parents, for example, are being increasingly called upon to make reproductive decisions based on ‘risk factors’ (rather than definitive diagnoses), and in relation to increasingly obscure conditions with uncertain prognoses (Novas and Rose, 2000; McClaren et al., 2008). It is against this backdrop of burgeoning probabilistic reprogenetic information, yet also an increased use of reproductive genetic technologies, that experiential knowledge has gained status as a tangible resource with which to navigate complex decisions that have uncertain outcomes (Boardman, 2014). The majority of studies that explore experiential knowledge in the domain of reproduction have focused on women’s embodied experiences of pregnancy and how these are brought to bear on decision-making (e.g. Lippman, 1999; Katz Rothman, 1984; Markens et al., 2010; Abel and Browner, 1998). However, more recently, ‘experiential knowledge of disability’ – that is, the insights born out of the daily realities of living with a disabling condition-has also been acknowledged as another form of experiential knowledge that may exist independently of, or co-exist with, women’s embodied experiences of pregnancy (France et al., 2011; Etchegary et al., 2008; Boardman, 2014, Boardman et al., 2017a).

‘Experiential knowledge of disability’, it is argued, is of particular relevance in the context of prenatal screening, testing and selective termination decisions as it may be used as a resource with which to imagine- and appraise-the nature and quality of future lives affected by that condition (Boardman, 2014; Dudding et al., 2000; Sawyer et al., 2006; Polnay et al., 2002; Raspberry and Skinner, 2011; France et al., 2011; Etchegary et al., 2008). For this reason, much like women’s bodily experiences of pregnancy, experiential knowledge of disability has been ascribed political value (Bricher, 1999; Parens and Asch, 2000; Asch and Wasserman, 2015) and regarded by many-particularly disability rights supporters-as the counter-weight to medicalised representations of disability in screening and testing contexts (Williams et al., 2002; Potter et al., 2008), offering alternative insights into life with the condition that are deemed to fall beyond the remit of reproductive genetic medicine (Ahmed et al., 2007).

Whilst this body of literature highlights the growing acknowledgement and various uses of ‘experiential knowledge of disability’ in reproductive contexts (Etchegary et al., 2008; France et al., 2011), it nevertheless remains a concept which is poorly defined and understood. Indeed, many commonly accepted understandings of experiential knowledge have cast it primarily in terms of its similarities and differences to medical knowledge, in order to either highlight its deficiencies (Prior, 2003) or to demarcate its contrasting areas of strength (Wynne, 1996). Whilst this comparison has been pivotal to the acceptance of experiential knowledge as a site of alternative expertise, however, this framing nevertheless also relegates the domain of the experiential to a state of perpetual dependence; as ‘always-in-relationship-to’ medical knowledge, ever vulnerable to critiques of deficiency, lack and inferiority.

This paper considers this position and the role and value of ‘experiential knowledge of disability’ as it is produced by and through accounts of reproductive decision-making. Drawing on 34 longitudinal in-depth interviews, the presented analysis explores the reproductive attitudes and decisions of 17 women clinically defined as ‘at risk’ of transmitting a neuromuscular condition, Spinal Muscular Atrophy (SMA). Two interviews were conducted with each participant (with a 6/7 year interval) in order to capture the shifting nature of their lived experience of SMA and to chart changes in their reproductive views and decisions during this time. By including two time points, and offering a comparison between them, this paper outlines the stark contrast between the cacophonous and ever-changing world of lived experience and the much more static and ordered realm of ‘experiential knowledge’. Finally, this paper will offer a critique of the notion that ‘knowledge’ is the appropriate prism through which to view and present experience. Through doing so, the various ways in which aspirations to knowledge status may paradoxically undermine, rather than bolster, the status of the experiential will be considered, highlighting the need for ongoing critical attention in this area.

### 1.1 Spinal Muscular Atrophy and reproductive genetics

After Cystic Fibrosis, SMA is the most common (potentially fatal) autosomal recessively inherited condition in the UK, meaning it is a single gene disorder requiring two carrier parents to transmit. SMA affects approximately one in every six thousand newborns in the North West European population (Dreesen et al., 1998). It is a neuromuscular condition characterised by generalised, and often severe, muscle weakness. SMA has been sub-categorised into distinct clinical ‘types’ (I-IV) with different presentations, ages of onset, severity of muscle weakness and prognoses ranging from early infantile death in the case of type I to adult-onset muscle weakness in adulthood in type IV (Dubowitz, 2008).

In order to understand the reproductive dilemmas faced by families living with SMA, it is firstly necessary to understand its typical mode of inheritance. It is estimated that between 1:40 and 1:60 of the general population are ‘carriers’ of SMA (i.e. they can transmit the condition but have no symptoms) (Wirth, 2000). When two carrier parents reproduce, they have a:

- 25% chance of a child who will have SMA.
- 50% chance of a child who will be an asymptomatic carrier.
- 25% chance of a child that will be neither a carrier nor have SMA.

Prenatal testing, Pre-Implantation Genetic Diagnosis (PGD) and cascade carrier screening (the genetic testing of family members of people with SMA) are all available on the NHS for families with a confirmed history of SMA.

## 2 Methods

Interviews were conducted at two time points.

### 2.1 Phase 1 (P1): in-depth interviews, 2007-9

The first round of interviews was conducted 2007–2009 as part of a larger study of sixty-one participants with SMA in their family (Boardman, 2010, 2014). The interviews were designed to elicit participants’ stories of life with SMA and a discussion of their views around, and (intended) uses of, reproductive genetic technologies. Participants were recruited through the main support group for families living with SMA in the UK-SMA Support UK. Participants were recruited using a variety of channels; through the group’s annual conference, advertisements in their publications, personal contacts and snowball sampling. Two participants were also recruited through disability organisations. The 17 participants whose data is reported on in this paper were all recruited through SMA Support UK.

Interviewing took place through a variety of channels (telephone, face-to-face and email), allowing for participant preference and practical constraints. Telephone and face-to-face interviews lasted, on average, for 70 minutes, and email interviews took place over periods lasting from three weeks to eight months. Email interviewing is a method of interviewing whereby interview questions and answers are exchanged electronically (Burns, 2010). This method of interviewing allowed participants to answer in instalments, at dates and times of convenience (McCord and Schwaber Kerson, 2006). Use of this method facilitated participation due to the potentially emotionally demanding nature of the interview and because the majority of participants were caring for young children and/or managing complex disabilities.

### 2.2 Phase 2 (P2): in-depth interviews, 2013-4

The second round of interviews took place 2013–14, some 6/7 years after P1. The second round of interviews was undertaken opportunistically as part of another research project. As P1 was not initially designed to be a longitudinal study, participants’ contact details were not stored beyond its completion. Advertisements were therefore placed in SMA Support UK’s newsletters again for previous participants to volunteer for a repeat interview. This second round of interviewing was designed to explore participants’ ongoing experiences with SMA, any reproductive decisions undertaken since P1 and any changes in views around, or uses of, genetic technologies. Informed consent was sought from each participant at P2, not only for a second interview, but also for the re-interrogation of their P1 interview beyond the originally intended timeframe.

The calls led to responses from 21 individuals. One individual was excluded as their diagnosis had changed since P1 and a further two because they had not participated in P1. One individual initially responded, but subsequent attempts to arrange an interview were unsuccessful, leading to a final sample of 17 individuals who were interviewed at both P1 and P2, all of whom were women and whose ages ranged from 25 to 61 at P2 (average age 39). As the interviews were designed to explore SMA experiences as well as reproductive decisions and attitudes, women who were no longer at reproductive age at P2 (defined as 15–44) were nevertheless still included. Four women were not of reproductive age by P2, although one of them was still within range for much of the P1-P2 time lapse. The range of experience with different types of SMA was broad, with the final sample including four participants with experience of type I, seven with type II, four with type III and two participants associated with variant forms of SMA, Spinal Muscular Atrophy and Respiratory Distress (SMARD) and Spinal Bulbar Muscular Atrophy (SBMA).

The sampling strategy for P2, whilst leading to the successful recruitment of 17 participants, posed particular limitations. Firstly, the sample was limited to those who self-selected for a second interview, which may reflect their desire to tell a particular story. Moreover, reliance on SMA Support UK to identify participants may have excluded individuals whose involvement with SMA Support UK had dwindled or ended entirely, or those who did not identify with the aims or ethos of the organisation. However, the participants who took part at P2 nevertheless represented a broad spectrum of experiences: eight had SMA themselves, five were parents, and the sample also included one aunt, two siblings and a spouse of someone with SMA. The sample also included two mothers whose child with SMA had died during the P1-P2 time lapse.

Fifteen of the P2 interviews were carried out over the telephone and two were carried out face-to-face, with the majority opting for the same method of interview as they had done in P1.

Ethical approval for the study was obtained from the Biomedical and Scientific Research Ethics Committee (Warwick Medical School).

## 3 Analysis

Interviews from both P1 and P2 were transcribed verbatim (with names and identifiers removed or changed), resulting in 34 interview transcripts (P1 and P2 combined). A single researcher conducted all of the interviews and qualitative analysis, under the supervision of two senior academics. As the P1 data had already been analysed in 2006–7 using a constructivist grounded theory approach (using Nvivo 7), the P2 data was imported directly into the existing Nvivo project file and the two transcripts were assigned to an individual participant using Nvivo 10’s ‘case’ function. An iterative comparative analysis was then undertaken which allowed for an exploration not only of participants’ ongoing stories and their reproductive decision-making, but also an in-depth analysis of the various ways in which the re-telling of their experiences-in light of later events-contrasted with P1. This was done using a comparative analysis approach and both using, and extending, the existing coding framework from P1, as well as cross-referencing emergent themes with key concepts from the relevant literature. Whilst the literature was used to interpret the developing analysis, however, the direction of the analysis remained ‘data driven’ throughout (Gibbs, 2007). For example, it became clear that there were divisions between those participants who had remained consistent in their attitudes and decisions between P1 and P2, and those who had changed their minds with growing experience and altering life circumstances. This division was critical to the development of the analysis.

The fact that the P1 data had already been analysed strengthened the approach to the secondary analysis as it meant that the researcher was blind to the unfolding narrative of the participants (due to the length of time that had elapsed). The independence of the two strands provided an analytic distance that facilitated a focus on the, often subtle, contradictions and discrepancies between the accounts given at the two time points, that might otherwise have been missed.

The women whose data are presented within this paper were selected because they most eloquently and clearly captured the key themes and findings of the analysis.

## 4 Results

The results of the analysis for this paper will be divided into two sections. The first section explores the views and decisions of women who presented their experiential knowledge of SMA as being of cumulative value over time. The second section highlights the perspectives of women who instead presented their experiential knowledge as existing in multiple (and sometimes competing) versions, although not necessarily of unequal value.

### 4.1 ‘I look back now and think I was quite naïve’: experiential knowledge as cumulative

Seven of the seventeen women in the sample compared and contrasted the nature and value of their experiential knowledge between P1 and P2. One such person, Melissa, aged 44 at P2, is the sister of Andrew, who was diagnosed with Spinal Bulbar Muscular Atrophy (SBMA) in his late twenties, during Melissa’s first pregnancy. Spinal Bulbar Muscular Atrophy, unlike classic SMA, is X-linked, which means it is transmitted by females and expressed in males. Given her brother’s diagnosis, Melissa had a 50/50 chance of being a carrier herself. The male children of female carriers have a 50/50 chance of developing SBMA, whereas the female children of female carriers have a 50/50 chance of being a carrier. Melissa described her reaction to her brother’s diagnosis and the perceived risk to her pregnancy in the following way at P1 (when her child was two years old):

After Andrew’s diagnosis, I was in fact some 10–12 weeks pregnant, had just split with my husband, the father of my baby, and to be honest hadn’t really given it [the possibility of SBMA affecting the foetus] much thought ... until my Doctor referred me urgently for a test to be carried out. I attended [hospital] and after a consultation I was told I could be screened to see whether I was a carrier via a blood test and I was also offered a test for the unborn child via taking fluid from the womb. Although it was in some ways a difficult decision, I discussed all the options with the doctor, and I didn’t feel too concerned. I came to the conclusion that I wouldn’t have the blood test and I would let life take the ‘fate test’ on the child, as it could be a girl and I believed “what will be will be”. I knew that the baby and I would cope with whatever happened, and I still believe that now. I have never believed in termination and so I think I would do the same again if I were to ever have another child.

(P1 email interview: Melissa)

Melissa ultimately decided not to have any testing for SBMA and went on to have a male child, Toby, six months later. At her P1 interview, Toby was two years old and Andrew had started using crutches to walk, but was still working. By P2, however, 6 years and 10 months later, life was very different for Melissa. Her son, Toby, was 8 years old and she had re-married. Andrew’s health had deteriorated significantly to the point that he used a wheelchair full time had given up work, and Melissa described him as ‘virtually house bound’. Melissa had become involved with attending to his personal care needs which she fitted around a part-time job and her son, a lifestyle which she described as ‘difficult to juggle’. Furthermore, Melisa’s nephew (her sister’s son), Mark (aged 18 at P2), had recently undergone genetic testing and whilst asymptomatic at the time, was found to have SBMA. As a response to this, Melissa took the decision to undergo genetic testing herself to determine whether Toby could develop SBMA. She was found *not* to be a carrier. Melissa described her decision in the following way:

It’s been a tough few years in a lot of ways. Mark’s [nephew] diagnosis hit us hard, and hit him hard. I see him looking at his uncle and wondering when that’s going to happen to him, you know, and it just .... breaks my heart actually. My sister is trying to encourage him [Mark] to take a career path that he can easily carry on with if he does end up in a wheelchair, but he wants to do carpentry, and so they’re having that battle now, so it’s already causing problems. And when I look back, I …. naively I suppose …. I mean I look back now and think I was quite naïve when I sat in that [hospital] when I was pregnant with Toby and I was just so full of happiness about my first baby, I wanted him so much and I just said, ‘oh no, well regardless of what, you know, whether it’s a boy or a girl, whether it has it [SBMA] or not I wouldn’t-test’, you know, I’d never get rid of it, and that probably *was* because it was my first child, my brother wasn’t really heavily affected at that point, it wasn’t, you know, the progression hadn’t really hit in ...[...]... I think having seen my nephew’s reaction …. that could be my son …. so I underwent the genetic test for him [Toby] and luckily it came back all clear. But I did feel guilty. I did. Horribly guilty for what I’d done. I thought, what a risk I took, what a gamble I took on your life. And I just didn’t even properly realise ... I don’t think .... that I was even taking it.

(P2 telephone interview: Melissa)

Melissa’s ongoing empathetic experiences with SBMA through her brother’s deterioration, her nephew’s diagnosis and then the birth of her own (male) child all contributed to a complete overhaul of her conceptualisation of SBMA between P1 an P2. Her desire to surrender control of whether her child had SBMA at P1 was, by P2, replaced with a new sense of responsibility to prevent the condition from ever reoccurring. While the genetic risk to Toby remained entirely unchanged between P1 and P2, Melissa’s estimate of it did. It was not until she experienced the ongoing realities of life with genetic disease, together with its reverberations through her family (in the form of her nephew’s diagnosis), that Melissa felt she appreciated the full pictures of what life is truly like with SBMA, and consequently, the implications of the decision she had made some eight years previously.

When Melissa conceived again, shortly after receiving confirmation of her non-carrier status, and 6 months prior to P2, she approached the pregnancy in an entirely different way, although the pregnancy ended in spontaneous miscarriage at 12 weeks:

It was just a very different experience [compared to first pregnancy]. I’m just a very different person now ... I didn’t have any testing at all with Toby, but this time I had everything I was offered. Unfortunately for us, the baby actually died before we had our results [standard antenatal screening tests]. My husband and I had made the decision that if anything showed up, we would have to seriously consider a termination. I wouldn’t *want* a termination, and I still don’t think I agree with it, but I suppose I don’t feel as prepared to make the call about what a baby should or shouldn’t have to endure in its lifetime, like I did before. I don’t think I can play God again. I thought I knew, and I think I got it wrong, because you don’t know how that child would handle it in their life. Mark and Andrew both suffer terribly, you know, in different ways, but they do suffer, and is it fair to put that onto someone? And to *know* you’re putting that onto someone? You have to think about it [the baby] living with that condition all its life-even after you’ve gone- and you know, things constantly change. In the end, the pregnancy didn’t go ahead, but at least this time I [sigh] ... I felt I’d done all I could.

(P2 telephone interview: Melissa)

Unlike her pregnancy with Toby, which Melissa described as being full of the happiness, expectation and novelty that a firsttime and much-wanted pregnancy brings, Melissa approached her second pregnancy as a ‘different person’, altered by the realities that genetic disease had brought upon her family and less certain of her ability to evaluate the point at which life became intolerable. It is noteworthy that through accounting for her dramatic turnaround between P1 and P2, Melissa presented her most recent experiences at P2 as a more accurate version of SBMA than those she presented at P1. The incorporation of Andrew’s deterioration and Mark’s pre-symptomatic diagnosis into her experiential knowledge relied on the invalidation of her earlier experiential knowledge even as it framed, and contributed to, her current decision to undergo antenatal screening. By presenting her experiential knowledge as being of cumulative value, and dismissing her earlier perspective as ‘naïve’, Melissa ‘over-wrote’ her own experiential knowledge to enable her to more easily navigate the complex terrain of reprogenetic decision-making and familial responsibility.

Whilst both Melissa’s experiential knowledge- and ultimately her reproductive decision-were completely reversed between P1 and P2 (as she experienced the increasing encroachment of SBMA on various spheres of her life), experiential knowledge emerged from her accounts as a pliable resource; malleable from both the inside (as her experiences of the condition shifted), but also vulnerable to external reconfiguration (by experiences entirely unrelated to SBMA).

As SMA is generally understood to be mildly progressive, it might be assumed that experiences of it become increasingly difficult overtime, however, this was not the case for all participants. Faye was aged 31 at P2, having been diagnosed with type II SMA when she was three. Faye has never been able to walk unaided, using an electric wheelchair from age five. At P1, Faye was 24 and struggling to find accommodation and work to suit her physical needs and was frustrated that as a young adult she was both financially and physically dependent on her parents. At P1, Faye was clear that she did not want to have children of her own, and that SMA was a key part of this view:

I personally am quite clear that I do not want my own children…. It would be hard for me as I know that any natural child of mine would inevitably inherit the SMA defective gene ... Whilst I am accepting that SMA is part of who I am, and I can mostly overcome a lot of the challenges because I live a very privileged lifestyle in lots of ways … there is no denying that it is not all sunshine and roses and can, at times, make things bloody complicated! I am just not sure that I feel comfortable knowing that I could be putting others at risk …. and continuing the line of this defect, which would be inevitable if I were to have children of my own. But if, for whatever reason, I did become pregnant, then yes I’d want to test them for SMA and I’d have to seriously consider what to do …. . I’d rather it stopped with me, you know?

(P1 telephone interview; Faye)

Whilst Faye recognized the critical role that her environment and ‘privileged lifestyle’ played in mediating her experiences of SMA, she nevertheless still regarded it as a ‘defect’ that she would not want to transmit to others. Kenen (1994) has referred to a sense of ‘genetic responsibility’ that emerges alongside the expansion of genetic knowledge. As we come to learn more about socially undesirable traits and propensities within our genetic make-up, a parallel sense of responsibility to prevent their transmission to future generations is also developing (Hallowell, 1999; Downing, 2005; Rapp, 1998). However, by P2, 7 years and 3 months following her P1 interview, Faye’s sense of responsibility was very different. At this point, Faye was living independently (with the support of paid assistants) and working full time in a job she loved:

…. I’m 31 now and although I still don’t want to have children, I’m suddenly surrounded by everyone else having children … Whilst that’s lovely, it has brought up … I guess it’s suddenly very real now. And I had a pregnant friend recently who was told she was at high risk of her baby having Down’s Syndrome ... and she, just in a really off-the-cuff comment said to me ‘well of course I’d get rid of it’ [if Down’s Syndrome confirmed]. And you know …. . that just really made my skin crawl, and I just didn’t know how to react … I found myself thinking about it more and looking at all of my friends thinking, ‘I’m disabled, how many of you would have gotten rid of me?’ You know, I don’t have Down’s Syndrome-but I have something else, and where do you draw the line? I realised in that moment that in my heart of hearts I don’t agree with it [testing and selective termination]… […]… When something forces you to look at your life in ‘life and death’ terms, I guess it made me think that …. I have a lot to offer, actually, in spite … or maybe even *because* of my SMA. Now I’m older and I’ve got a bit more life experience I can say that with some certainty. There are far worse things that could happen to you than having SMA, you know? It’s no tragedy.

(P2 telephone interview; Faye)

It is noteworthy that while Faye reported that her physical symptoms of SMA had altered somewhat over the time lapse between interviews, that it was instead her changing relationship to parenthood, her direct confrontation with selective reproduction, her increased life experience and altered living/ working environment that contributed most to her reevaluation of SMA. Through her empathetic experiences of her friend’s pregnancies (Abel and Browner, 1998), Faye’s sense of genetic risk-previously ‘latent’-had been brought sharply into the realm of the ‘manifest’ (Parsons and Atkinson, 1993). Unlike Melissa, whose experiences of SBMA deterioration were central to the revision of her experiential knowledge, Faye’s account highlights the way in which events seemingly unrelated to SMA could prompt a revision of her views of SMA and attitudes towards selective reproduction.

While emanating from contrasting sources of lived experience (both within and without SMA), the complete turnaround of both Faye and Melissa’s experiential knowledge between P1 and P2 were part of a similar approach to distillation. ‘Distillation’ refers to the process of ordering and filtering lived experience into a comprehensive body of knowledge (Bulme, 2016). For Melissa and Faye, this ordering involved the construction of experiential knowledge as incrementally valuable, accumulating and retaining status in a linear manner. With this approach to distillation, Faye and Melissa’s P2 accounts not only cancelled out, but actually replaced their P1 viewpoints in a continuous cycle of quasi-paradigmatic shifts. For Faye and Melissa, as well as the other five women who generated and used their experiential knowledge in this way, the domain of the experiential could be directly transposed onto the epistemic framework of knowledge, a framework which esteems high quantities of cumlative information and disvalues contradictions.

Not all participants, however, strategically invalidated their previous perspectives in order to bolster the authenticity of their current views. For these participants, the evolution of experiential knowledge through time was more nuanced, with several contrasting versions of it co-existing simultaneously. It is to these accounts that I will now turn.

### 4.2 ‘The pain is still there, I’m just looking at it from further away’: experiential knowledge as fragmentary

For ten of the 17 women included in this analysis, the passage of time between P1 and P2 did not bring about a perceived increase in the amount, and value, of experiential knowledge as for Faye and Melissa, but rather brought into critical relief the significance of context in the formulation of fragmentary and competing versions of experiential knowledge.

Annette was 30 years old at P1, working part-time as a solicitor and living with her husband, Simon, on the east coast of England. At P1, Annette was going through a traumatic time, having experienced the death of her infant daughter, Scarlett, to type I SMA (aged 14 months) just 3 months previously. Annette described her experiences and views on SMA and reproduction:

You see, I think SMA is just a devastating condition. Our little Scarlett was as bright as a little button, full of life and full of smiles right until the end really…. you know, her mind was …. it was her body that gave out on her, and really, that’s the cruelty of SMA. These kids are often intelligent, they’re …. mentally, they’re intact, as it were. And to lose your child to something like that, well to say it’s any parent’s worst nightmare would be the understatement of the year I think! I made my mind up as she died, as I held her, that that would be it for me, no more babies. No more SMA for us. I wouldn’t …. I *couldn’t* put another baby through what our Scarlett went through.

(Annette, face-to-face interview P1)

With the trauma of losing her young daughter still raw, Annette was clear at P1 that her experiences would not be repeated. Her sense of responsibility was both underscored and clarified through her experience of Scarlett’s death, extending backwards and forwards in time and incorporating her felt sense of duty not only to Scarlett, but to imagined future children as well as to herself.

Annette’s P2 interview, in contrast, took place shortly after her 37th birthday, 7 years and 8 months after P1. By this time, she was working full time and described her life as ‘full’ and ‘chaotic’. Since P1 she had become heavily involved with charity fund-raising (primarily for SMA-related charities) and offered her weekends and evenings to support families affected by a type I SMA diagnosis. Annette reflected on this during her interview:

You see, even after Scarlett’s death, SMA is very much still part of our lives. It’s still with us, and I honestly think that’s a good thing as it’s made us better people. I’m much more understanding now than I was back then, I give a little more, if you see what I mean. I’m less judgmental. I’ve learnt new skills. You know, I talk to people about what happened to us, I tell Scarlett’s story. That’s something I could never have done [previously] as I’m actually a very private person. And that’s all down to Scarlett really ...[..].. I did say I wouldn’t have another SMA baby, and I haven’t, but I suppose I’m less judgmental about that now …. . I try and focus on all the positives that Scarlett brought into our lives and continues to do, and I’m grateful every day that she lived. I’m grateful for every day of those 14 months, and that’s changed, because I couldn’t think like that before. I couldn’t bring myself to be grateful, I could only see what I’d lost, not the wonderful gift I’d been given, and that gift was her.

(Annette, telephone interview, P2)

It has been widely observed in the literature that bereaved parents must navigate not only the grief and loss at the death of their child, but must also re-construct their identities and lives in the liminal and ambiguous spaces that emerge between parenthood and childlessness (Young et al., 2002). Whilst still resolute that her reproductive decision had not, and would not, change, nevertheless, her framing of this, both with and through her experiential knowledge of SMA (‘telling Scarlett’s story’), had entirely transformed. Annette was now able to focus on the positive aspects of Scarlett’s short life, changing her narrative of grief and loss into an instrument of healing and hope, and one that Annette was using purposefully to both ease the pain of others and preserve her daughter’s legacy.

Abel and Browner (1998), by focusing on women’s various experiences of pregnancy and of caring for relatives with Dementia, have highlighted the different forms of experiential knowledge that emerge out of embodied and/or empathetic experiences with a phenomenon. According to their conceptualization, experiential knowledge emerges directly out of visceral and/or emotional encounters with the world, such that there is a clear and direct mapping between lived experience and the ways in which this comes to be translated, or ‘distilled’ into experiential knowledge (Bulme, 2016). Annette’s account, however, highlights that this process of distillation between ‘experiencing’ and ‘knowing’, was not necessarily one of simplification and reductionism. Rather, Annette acknowledged contrasting versions of Scarlett’s life and death that existed as she selectively privileged different aspects of the story at different points in so far as they resonated with her present reality:

Doing the charity work that I do now, I do go over it [Scarlett’s story] quite a lot [with newly diagnosed families].…and even though I’m …. I’m in a very different place now, I don’t sugar coat it for them, you know? I tell them exactly how … it was . and depending on what day they get me on, they may hear more or less of the positive side of it [laughs]. Sometimes, like around her birthday, I tell it very differently … but grief’s like that isn’t it? It ebbs and it flows, but to be honest, I don’t try to make them ‘see the bright side of it’ anyway, because actually that’s the reality of it. I think it’s important they can be negative about it if they want to be. You know, I am only able to be the way I am about it because I’m no longer in the eye of the storm, as it were, and the fact that I can see they will come out the other side tomorrow doesn’t take away how painful it is for them today ...[...]... and it’s the same for me, the pain is very much still there, I’m just looking at it from further away.

(Annette, telephone interview, P2)

Unlike Faye and Melissa, Annette’s P2 interview and her selfidentified recovery from being in the ‘eye of the storm’ did not render her experiential knowledge of this time of less value than her current version of it. Annette told different, but equally accurate, versions of Scarlett’s life and death in response to her current circumstances and positionality. Callon and Rabeharisoa (2002) as well as Pols (2013) have written about the fluid characteristics of experiential knowledge that are often overlooked in the literature. They highlight the dispersion of experience into multiple- and sometimes competing-bodies of knowledge across sources and sites. As Pols has argued in her study of COPD, patients pieced together their experiential knowledge from various sites including ‘translated bits of medical knowledge with homegrown know-how and tips from the neighbors with the weighing of different values in each new situation’ (Pols, 2013: 88). For participants such as Annette, the passage of time presented an additional site; her experiential knowledge at any moment was both co-produced and shaped by the very lived experience from which it emerged. Importantly, however, and in contrast to Faye and Melissa, Annette acknowledged that the fragmentary nature of her experiential knowledge was not its weakness, but rather its key strength, enabling her to ‘tailor’ her support to other bereaved parents by aligning her bereavement experiences with theirs.

## 5 Discussion

As the capacities of genetic technologies advance, reproductive decision-making is becoming increasingly divorced from the everyday lived experience of genetic disease. Population-level genetic screening programmes, for example, bring the notion of genetic risk into the lives of people with no previous association with, or experience of, genetic disease (McClaren et al., 2008); severing genetic disease experience (experiential knowledge) from the reproductive decisions about them. It is in this context that the need to understand the role and value of ‘experiential knowledge of disability’ is becoming increasingly important (Boardman et al., 2017b).

By drawing on qualitative interview data on reproductive decisions and attitudes with people at risk of transmitting SMA undertaken at two time points (6/7 years apart), this study explicitly demonstrates the complex relationship that people living in families affected by genetic disease have to their experiential knowledge and its uses within reproductive decision-making. By delineating the contrast between ‘lived experience’ (the constantly shifting realm of everyday experience) and ‘experiential knowledge’ (the ordered and re-countable version(s) of that lived experience), this study highlights that the process of distillation is both highly political and context dependent.

For seven participants, their distillation supported a conceptualization of experiential knowledge as constantly being overwritten. According to this perspective, experiential knowledge expands, and crucially-becomes more *authentic-* overtime and with increasing quantity. For participants who described their experiences in this way, the multiple (and competing) versions of their experiential knowledge that developed came to be strategically ordered and prioritized in order to add epistemic weight to current views and decisions (‘I can say that with some certainty’, Faye) and invalidate previous ones (‘I think how naïve I was..’, Melissa). This paradoxical conceptualization of experiential knowledge, at once reliable but also continuously in a sate of flux, change and incompleteness, vulnerable to changes both within and without itself, appeared at the heart of these women’s accounts. Indeed, it is noteworthy that all seven participants who fell within this category (including Melissa and Faye), described contrasting reproductive views and decisions at P2 than those expressed at P1, suggesting that this particular means of generating and presenting experiential knowledge was critical to their ongoing justification and accounting for, reversed reproductive views and decisions over time.

However, not all participants hierarchically ordered the different versions of their experiential knowledge. For the remaining ten participants, all of whom remained consistent in their reproductive views and decisions between P1 and P2, experiential knowledge emerged not as an incrementally valuable resource, but rather as a multiplicity of situated knowledges across time and context. For these participants, life with SMA could be told and re-told at various points and in contrasting ways without the need for one interpretation to be voided before the next could take its place. For Annette, for example, the interpretation and reinterpretation of Scarlett’s death at P1 and P2 produced two very different accounts of the same event from the iterant vantage point of her (ongoing) recovery. The multiple sites of her experiential knowledge did not render her previous accounts invalid, rather, these versions were instead incorporated into the complex and shifting picture Annette developed of her SMA bereavement.

Experiential knowledge emerges from this analysis, therefore, as containing multiple, competing and sometimes contradictory frameworks for understanding and appraising genetic disease which are often strategically prioritized or invalidated in the context of reproductive decision-making. Given this complexity, the possibilities (as purported by disability rights supporters) for usefully incorporating it within reprogenetic decision-making appear limited. Indeed, its demonstrated divergence from the epistemological and ontological assumptions of ‘knowledge’ have been regarded as rendering it an inappropriate resource with which to make significant health-related decisions (Prior, 2003). However, to dismiss the value of experiential knowledge based on its ability to simulate, or replace, medical knowledge is to misconstrue its value (Pols, 2013). Reprogenetic decisions are not approached in isolation through the weighing, sifting and appraising of medical information alone, but rather are highly complex and multi-faceted decisions to which multiple sources and sites of knowledge and information are brought to bear (France et al., 2011). Assigning the label of ‘knowledge’ to experiential insights into disability while once serving an important, and politically expedient, function in bolstering its status vis-à-vis medical knowledge, has led to the persistent framing of experiential knowledge by and through its relationship to medical knowledge. In an age where the fallibility of medical knowledge is increasingly being acknowledged, however, and traditional power boundaries between patients and the medical profession re-configured, the political impetus to maintain this dichotomy has greatly diminished. Indeed, even though medical and experiential knowledge invariably feed into one another in various ways (Markens et al., 2010), the insights into disability possessed by the people who live intimately with it is inherently different in character, substance and form than medical knowledge. By valuing experiential knowledge on its own terms, independent of the rubric of knowledge and expertise, we may more usefully be able to justify and harness its incorporation into reprogenetic decision-making, without the need for misleading claims about its permanence, stability and universality. Indeed, it is these very areas of divergence from ‘knowledge’, its constant oscillations and state of flux as a ‘living’ resource, that the realm of experiential draws its key strength.
